# Evaluation of Polymethyl Methacrylate as a Provisional Material in a Fully Digital Workflow for Immediate-Load Complete-Arch Implant-Supported Prostheses over Three Months

**DOI:** 10.3390/ma18030562

**Published:** 2025-01-26

**Authors:** Luis Carlos Garza, Eduardo Crooke, Marta Vallés, Joan Soliva, Xavier Rodríguez, Mariona Rodeja, Miguel Roig

**Affiliations:** 1 Department of Restorative Dentistry, Universitat Internacional de Catalunya, 08195 Barcelona, Spain; luiscarlos@uic.es (L.C.G.); mvalles@uic.es (M.V.); jsoliva@uic.es (J.S.); marionarodeja@uic.es (M.R.); mroig@uic.es (M.R.); 2 Private Practice, 29016 Málaga, Spain; 3 Department of Oral Surgery, Universitat Internacional de Catalunya, 08195 Barcelona, Spain; rodriguez@borgbcn.com

**Keywords:** polymethyl methacrylate, interim complete-arch prostheses, fully digital workflow, marginal bone loss

## Abstract

While complete-arch digital-implant-scanning protocols have been described, their clinical outcomes when using polymethyl methacrylate (PMMA) as a provisional material remain insufficiently substantiated. This clinical study aims to integrate digital solutions into implant dentistry and establish PMMA as a reliable material for immediate-loaded protocols. Fifty-six patients received 432 implants and 72 immediate fixed interim complete-arch prostheses, all fabricated using a fully digital workflow. Patients were followed up at 3 months to evaluate implant survival, prosthesis survival, and implant mean marginal bone loss using an interim PMMA prosthesis. Patients completed the Oral Health Impact Profile (OHIP) questionnaire to determine the implants’ impact on quality of life. Statistical analyses included analysis of variance, Fisher’s least significant difference (LSD) test, and the Wilcoxon signed-rank test. Of the 432 implants placed, only two failures were observed. Marginal bone loss (MBL) was significantly greater in male patients (*p* = 0.002) and older smokers (*p* = 0.016). Patient-reported outcomes, as measured by the OHIP questionnaire, demonstrated significant improvements in quality of life. PMMA is a reliable material for immediate-loading protocols in fixed interim complete-arch implant prostheses. Its combination of mechanical strength, biocompatibility, and esthetic properties, along with the accuracy of fully digital workflows, ensures predictable clinical outcomes.

## 1. Introduction

Biomaterials are at the forefront of innovation in implant-supported prostheses, providing solutions that combine functionality, esthetics, and biocompatibility. Among these, polymethyl methacrylate (PMMA) has emerged as a key material in dental applications, particularly for interim prostheses, which are used in immediate-loading protocols [1]. Its combination of mechanical strength, esthetic versatility, and cost-efficiency makes it a preferred option, especially when paired with digital workflows that streamline treatment planning and execution [2]. 

PMMA’s applications extend beyond dentistry to include uses in orthopedic implants and craniofacial prosthetics, where its biocompatibility and mechanical properties have proven invaluable. Recent studies have demonstrated its superiority in maintaining structural integrity under high stress, supporting its versatility in medical contexts.

Nowadays, PMMA is a versatile polymer commonly used in dentistry, especially in prosthodontics. Its applications include artificial teeth, denture bases, and temporary crowns [3]. The popularity of this material results from its necessary properties, such as esthetics, cost-effectiveness, and ease of manipulation [3,4].

However, PMMA has limitations, including susceptibility to hydrolytic degradation and inadequate fracture toughness [4]. To confront these issues, recent research has concentrated on developing PMMA-based nanocomposites by incorporating various nanofillers like metallic nanoparticles, metal oxides, and carbon-based nanomaterials. These modifications seek to enhance PMMA’s properties, including resistance to water absorption, thermal stability, and flexural strength [3,4].

The introduction of digital workflows in implant dentistry has transformed traditional approaches, delivering exceptional accuracy, efficiency, and consistency [5]. This paradigmatic shift has driven the adoption of monolithic materials like PMMA, which facilitate the integration of digital workflows into prosthetic fabrication while enhancing biomechanical performance. PMMA, in particular, stands out for its strength, biocompatibility, and esthetic qualities, making it an excellent candidate for immediate-loading complete-arch interim prostheses.

Despite its growing popularity in restorative dentistry, clinical studies evaluating PMMA in high-demand applications such as complete-arch, immediate-loading implant-supported interim prostheses are scarce [6]. This lack of comprehensive evidence has hindered its widespread acceptance for treatments requiring functional durability and esthetic excellence. Evaluating its reliability, particularly in terms of marginal bone loss (MBL) [7], implant and prosthesis survival rates [8], and patient-reported outcomes [9], is crucial for expanding its clinical applications.

The immediate loading protocol, where a prosthesis is placed within one week of implant surgery, demands materials that can endure early functional loads while supporting successful osseointegration [10]. Milled PMMA has demonstrated superior flexural strength and fracture resistance [11] compared to conventionally processed alternatives, making it a suitable option for these protocols [12]. Recent studies also highlight its positive impact on oral health-related quality of life (OHRQoL), further reinforcing its clinical value [13].

This clinical study aims to address this gap by evaluating the clinical performance of PMMA as a provisional material in complete-arch, implant-supported rehabilitations using a fully digital workflow. By assessing implant and prosthesis survival rates, marginal bone loss, and patient outcomes in a prospective cohort, this research provides critical evidence supporting the integration of PMMA into immediate-loading protocols. These findings not only validate its role in digital dentistry but also contribute to advancing the application of biomaterials in complex clinical scenarios.

## 2. Materials and Methods

This clinical study included seventy-two patients, all presenting with either complete or partial edentulism. Each patient required the extraction of their remaining teeth, followed by immediate implant placement. Ethical approval was granted by the Ethics Committee, and the research adhered to the principles of the Declaration of Helsinki regarding clinical research involving human subjects. All participants were provided with written informed consent for both the implant placement and study participation. The study was registered at ClinicalTrials.org (NCT05924451). 

The patients consisted of 36 men and 36 women, ranging in age from 33 to 84 years (mean age of 59.89 ± 10.6 years). The patients were selected based on the presence of terminal dentition in the maxilla or mandible requiring tooth extractions for full-arch implant-supported rehabilitation. Exclusion criteria were kept minimal, focusing on severe systemic conditions like uncontrolled diabetes or osteoporosis, which could impair osseointegration. 

Before treatment, all patients completed the Oral Health Impact Profile (OHIP-14) questionnaire to assess the impact of their oral condition on quality of life. The OHIP-14 evaluates key dimensions such as functional limitation, pain, discomfort, and disabilities across 14 items, each rated on a scale from 0 to 4 (0 representing optimal quality of life and 4 indicating poor quality of life). At three months post-surgery, the same survey was re-administered to evaluate changes in quality of life. 

**Figure 1:**
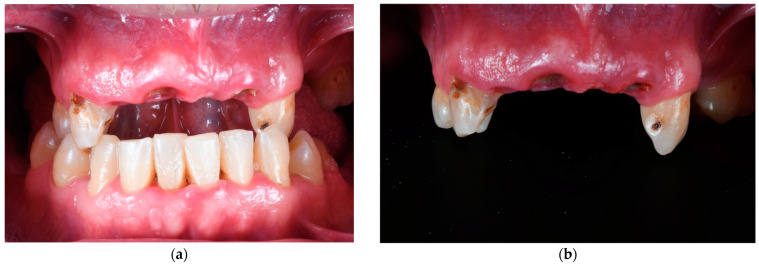
Initial photographs of an illustrative case. (**a**) Intraoral view of the initial view in maximum intercuspidation; (**b**) Frontal view of the upper dental arch.

Treatment planning followed the workflow described by Espona et al. [14]. Initial reference data included clinical photographs, as shown in the illustrative case in Figure 1, parallelized periapical radiographs, a digital intraoral scan exported as a standard triangle tessellations (STL) file, and cone beam computed tomography (CBCT) scans using i-CAT FLX unit (Imaging Sciences, Hatfield, PA, USA).

Implants were placed using prosthetically guided planning and the manufacturer’s recommended drilling protocol, aided by a surgical guide. Nobel Parallel Ti-Ultra implants (Nobel Biocare AB, Göteborg, Sweden) were used for all patients. These threaded, endosseous implants, fabricated from commercially pure grade IV titanium, feature a multi-zone surface with a gradual topographical shift from collar to apex. Implants were placed into either healed bone or extraction sockets using a one-stage surgical technique, with a minimum insertion torque of 35 Ncm required. 

Following implant placement, multi-unit abutments (Nobel Biocare AB, Göteborg, Sweden) were inserted and torqued to 35 Ncm per the manufacturer’s recommendations. Scan bodies (Avinent^®^ Implant System, Santpedor, Spain) were attached and tightened to the transepithelial abutments, and a post-operative IOS was made and exported as an STL file. Subsequently, a CBCT scan was made with the scan bodies in place, and the resulting dataset in the digital imaging and communications in medicine (DICOM) format was converted to an STL file.

The scan bodies were then removed, and healing caps were placed on the abutments. The laboratory received both the intraoral scan and the CBCT files. The STL files from the intraoral scan were corrected using the CBCT data to improve accuracy and alignment with the implant sites before initiating the prosthetic design.

**Figure 2:**
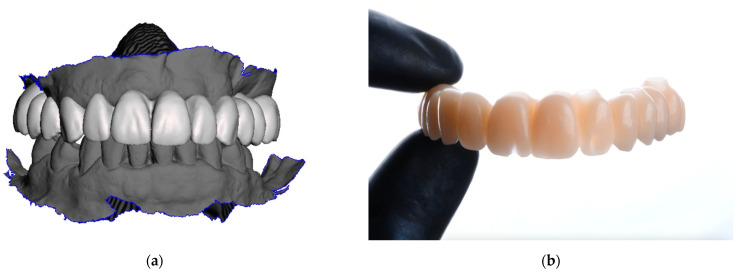
Immediate fixed interim PMMA complete prosthesis. (**a**) Frontal view of the digital design; (**b**) frontal view of the prosthesis delivered by the laboratory.

Using the corrected file, an interim PMMA prosthesis (Figure 2) was designed and milled from a PMMA disk (Huge Dental Material Co., Ltd., Shanghai, China) with a milling machine (DWX-52D^®^, DGSHAPE Corporation, Hamamatsu, Japan). Key design considerations included ensuring passive fit, optimizing occlusal contacts, and eliminating cantilevers to reduce stress on distal implants. The digital framework was virtually articulated to verify occlusal harmony and vertical dimension. Once finalized, the design was milled from a pre-polymerized PMMA block using a milling machine. Post-milling adjustments were made to refine the contours and ensure an optimal fit before clinical delivery. The prosthesis was delivered within one to two days after the surgery was completed. 

**Figure 3:**
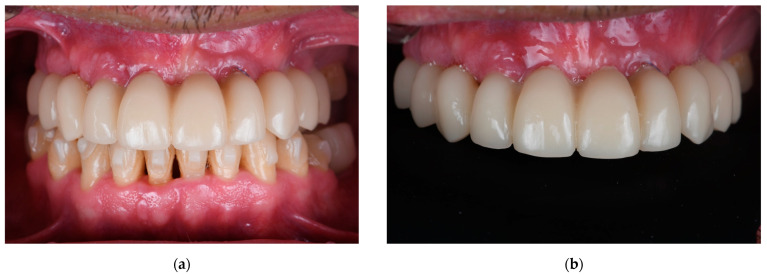
Three-month follow-up of the immediate fixed interim complete prosthesis using a fully digital workflow. (**a**) Intraoral view in maximum intercuspidation; (**b**) frontal view of the upper arch.

Clinical photographs, as shown in Figure 3, intraoral scans, and parallelized periapical radiographs were obtained post-delivery to assess the fit and marginal bone levels around the abutments. The fit was evaluated following the criteria of Roig et al. [15], which rely on tactile feedback when tightening prosthetic screws to judge passivity. 

Post-operative care instructions advised patients to adhere to a soft diet. Follow-ups were conducted 48 h and 3 months post-prosthesis delivery to monitor occlusal contact distribution by occlusal adjustments, ensure the passive fit of the prostheses, and remind patients of post-operative care guidelines.

The primary outcome variables assessed were implant success, marginal bone loss (MBL), and prosthesis fractures. Implant success was defined by implant stability, evaluated through manual torque testing during follow-up visits; the absence of radiolucency, with no evidence of radiolucent zones around the implants during the 3-month period; and mucosal suppuration or pain assessed clinically at follow-ups. Factors such as smoking, sex, implant platform, patient antagonist, and the number of implants per patient were analyzed.

Success criteria for implants were based on the modifications of Spiekermann and Jansen’s guidelines: no implant loss and no MBL greater than 4 mm at the mesial or distal aspects, as measured on periapical radiographs [16]. Prosthesis survival was defined as the prosthesis remaining functional without irreparable fracture, mobility, or patient-reported pain. Prosthesis failure was recorded if the prosthesis was removed for any reason. The cumulative survival rate (CSR) was calculated as the number of implants and prostheses surviving without failure during the study period, divided by the total number placed, expressed as a percentage.

MBL was measured from radiographs made post-surgery (T0) and at the 3-month follow-up examination (T1). The change in MBL from T0 to T1 was then calculated.

Prosthesis fracture was defined as any provisional prosthesis that became non-functional due to extensive implant loss and required removal. Implant failure rates were analyzed according to the following variables: smoking status, implant positioning, implant diameter (3.75 mm, 4.3 mm, or 5 mm), and antagonist material (complete resin, natural teeth, ceramic, or zirconia). 

Statistical analyses were performed using Statgraphics Centurion XIX (Statgraphics Technologies Inc., The Plains, VA, USA) software. Analysis of variance (ANOVA) methodology was applied to evaluate the differences in marginal bone loss (MBL) across various factors, and Fisher’s LSD test was used for post hoc comparisons. Before conducting ANOVA and Fisher’s LSD tests, assumptions of normality and homogeneity of variances were verified using the Shapiro–Wilk and Levene’s tests, respectively. No significant violations were detected, ensuring the reliability of the analyses. Wilcoxon’s signed-rank test assessed paired non-parametric data, such as pre- and post-treatment OHIP-14 scores. A *p*-value of <0.05 was considered statistically significant.

## 3. Results

A total of 56 patients participated in this study, resulting in the analysis of 72 prostheses and 432 implants. Table 1 provides an overview of clinical data, sociodemographic data, and implant-related variables.

Out of the 432 implants placed, only two failed—one in the maxilla and one in the mandible—occurred, indicating no statistically significant impact of implant location on the failure rates. Additionally, no significant differences were observed between smokers and non-smokers (*p* = 0.306). However, MBL showed a significant difference between sexes, as shown in Table 2, with men experiencing higher MBL than women (*p* = 0.002).

Age and smoking status appeared to influence MBL. Among non-smokers, no statistically significant differences were observed across the three age groups. In contrast, within the smoking group, individuals over 60 years demonstrated significantly higher bone loss rates (*p* = 0.016).

**Figure 4:**
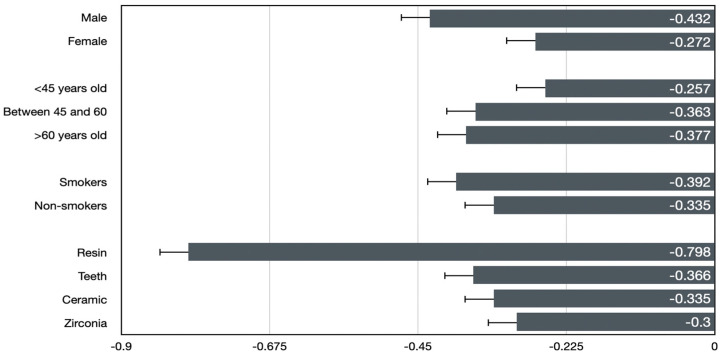
Mean MBL between variables.

Further analysis revealed no statistically significant differences in MBL with respect to implant diameters (*p* = 0.616). However, MBL varied significantly across the antagonist materials, with the highest MBL observed in complete resin antagonists and the lowest value in zirconia, as shown in Figure 4. Although the number of implants per patient did not show a statistically significant effect on MBL overall, range tests indicated some variability between groups with different implant counts.

**Figure 5:**
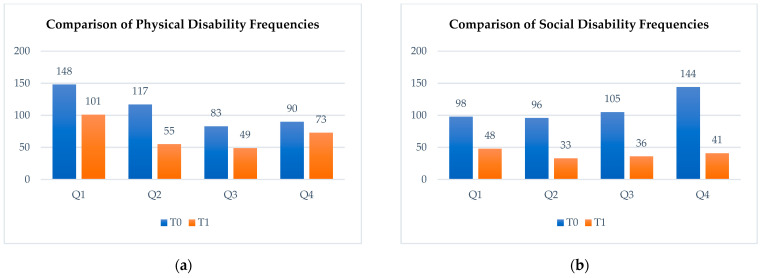
The Oral Health Impact Profile survey. The bar diagram illustrates the frequency of the following: (**a**) physical disability before treatment and at the 3-month follow-up; (**b**) social disability frequencies assessed before treatment (T0, shown in blue) and at 3-month follow-up (T1, shown in orange). Q1, Q2, Q3, and Q4 represent the first, second, third, and fourth questions, respectively, from the corresponding section of the OHIP-14 questionnaire.

Pre-treatment OHIP-14 results highlighted the significant impact of malocclusion, psychological discomfort, physical disability, and social disability on quality of life. Three months post-treatment, a second OHIP-14 survey revealed notable alleviations in these areas, as shown in Figure 5. 

## 4. Discussion

This study validated the durability of PMMA interim prostheses, the implant survival rates, and marginal bone loss within the protocol framework for complete-arch implant-supported rehabilitation using a fully digital workflow. The primary objective was to contribute to the growing understanding of factors influencing the success and failure of these prostheses, with a particular focus on immediate implant loading and digital workflows. 

We observed a 99.5% implant survival rate and a 100% prosthesis survival rate. Only two implant failures occurred—one in the mandible and one in the maxilla—during the first three months of osseointegration. These findings align with existing studies, which also report high survival rates for implants placed under immediate functional loading in complete-arch fixed prostheses, regardless of where they are placed in fresh extraction sites or healed sites [17]. The CSR observed in this study is consistent with immediate loading protocols, which report values between 95% and 100% for the edentulous mandible [18,19,20,21].

Several key factors contributed to the optimal survival of the implants and the prosthesis survival rate. These included the achievement of a passive fit for the PMMA prosthesis, the proper distribution of occlusal contacts, adherence to a soft diet during the initial post-surgical period, stringent oral hygiene practices, and the absence of cantilevers in the prosthetic design [22]. For this study, assessing passive fit involved a tactile evaluation, while the tightening of screws involved a visual and radiographic examination during screw placement using the Sheffield test [23]. Additionally, occlusal contacts were limited exclusively to centric contacts [24].

The impact of cantilevers on stress distribution in full-arch rehabilitations has been investigated in several studies. Cantilever length affects stress distribution [25]. The maximum stress values are typically found at the neck of distal implants [26]. Mandibular flexure can also contribute to stress build-up in distal implants and the prosthetic superstructure, particularly at the symphysis [27]. Overall, the interplay between implant inclination, cantilever length, and mandibular flexure significantly influences stress distribution in full-arch rehabilitations.

The survival rate of the prostheses can be attributed to the protocol’s meticulous verification, ensuring high accuracy in both prosthetic fit and occlusion, as well as maintaining the correct vertical dimension. This outcome contrasts with earlier studies reporting fracture rates of 3% to 10% for provisional prostheses on immediately loaded implants [28,29,30,31].

Recent research has investigated the application of milled PMMA for implant-supported full-arch interim prostheses. Milled PMMA has been shown to offer significantly greater flexural strength—35% higher than conventional heat-processed in both standard and cantilever configurations [1]. These prostheses have proven to be reliable, with some remaining functional and free of complications for up to two years [32,33].

Furthermore, the computer-aided design/computer-aided manufacturing (CAD/CAM) of milled PMMA exhibits a greater elastic modulus, ultimate strength, and toughness [34]. Regarding occlusal precision, 3D-printed crowns exhibited better dimensional accuracy than their milled counterparts [35]. However, milled PMMA provisional crowns displayed significantly reduced microbial adhesion compared to conventional materials over time, with both groups experiencing a decline in the colonization of Streptococcus mutans [36]. A practice-based study reported a 76% survival rate for milled PMMA interim prostheses after 12 months, alongside notable improvements in patients’ oral health-related quality of life [37].

The integration of computer-aided design and surgical planning has been identified as essential for achieving superior esthetic and functional outcomes in complete-arch PMMA restorations [12,37]. Collectively, these findings emphasize milled PMMA as a durable and patient-focused material for implant-supported interim prostheses.

PMMA-based materials for interim prostheses interact complexly with the oral environment, raising concerns about potential adverse effects [38]. However, PMMA has shown promising results in various applications. In infected knee arthroplasty, PMMA spacers with antibiotics have been effective in preventing soft tissue fibrosis and recurrent infections [39,40]. For full-arch implant-supported rehabilitations, in terms of soft tissue integration, direct PMMA abutments performed similarly to titanium and zirconia abutments in a mini-pig model, showing comparable soft tissue dimensions and peri-implant bone remodeling [41].

Regarding intraoral scanners (IOSs), the accuracy of IOSs is particularly critical in complete-arch scans for edentulous patients. Studies have highlighted significant differences in trueness, with values <193 μm for edentulous scans compared to values <150 μm for dentate scans [42]. This discrepancy raises concerns about the adequacy of digital impressions for ensuring the optimal fit required for complete-arch implant-supported fixed prostheses. Espona et al. [14] described a method for enhancing the accuracy of digital impressions using IOSs, which has proven effective in reducing the risk of biological and mechanical complications.

In this study, potential inaccuracies in intraoral scanning were mitigated by correcting the STL files with CBCT data. This technique improved the alignment and verification of implant positioning relative to the surrounding anatomical structures. However, alternative correction methods, such as the Medicalfit device, have shown equal efficacy in improving scanning accuracy [43]. Although CBCT remains a reliable modality for verifying implant positioning, limitations persist in ensuring the passivity and overall fit of the final prosthesis [44]. Additionally, CBCT image quality limitations may affect the precision of the prosthetic outcome [45].

Recent studies have compared photogrammetry (PG) systems with conventional methods for 3D dental implant positioning and facial scanning. PG systems demonstrated comparable accuracy to traditional techniques, with trueness ranging from 10 to 77 μm and precision from 2 to 203 μm for dental implant positioning [46]. For facial scanning, PG showed mean differences of 1.10 to −1.74 mm from direct anthropometry [47]. When compared to CBCT, 3D photogrammetry exhibited higher linear accuracy in patients with facial deformities, though it was more affected by protuberances [48]. However, some studies have questioned that accuracy and further research is needed to strengthen the evidence regarding the accuracy of the various commercially available PG systems [44].

By adhering to these protocols, non-passive fits and cantilevers were effectively eliminated, contributing to the 100% survival rate of the prostheses. Similar survival rates, ranging from 99.4% to 98.5%, have been reported by Coskunses et al. [49] in complete-arch implant-supported prostheses using a combination of narrow and standard-diameter implants.

MBL, a critical indicator of implant success, was also evaluated. We observed an MBL of 0.480 ± 0.628 mm after 3 months, which is lower than the typically reported 1 mm to 1.5 mm MBL within the first year of functional loading [50,51,52]. Although the shorter follow-up period in our study may explain the reduced MBL, our findings align with those of Coskunses et al. [49], who reported 0.63 mm of MBL.

Smoking has been identified as a significant risk factor for increased MBL in patients undergoing immediate functional loading protocols [18,53]. While the present study did not reveal statistically significant differences between smokers and non-smokers, smokers exhibited slightly higher MBL (0.392 ± 0.045 mm) compared to non-smokers (0.335 ± 0.033 mm). This trend is consistent with previous research [31].

Another critical factor in preventing MBL is patient hygiene, with poor plaque control linked to increased bone loss. The proper design of fixed restorations [54,55], allowing easy access for cleaning, is essential for maintaining healthy peri-implant tissue. The combination of effective oral hygiene instruction, plaque control, and accurate prosthetic fit played a significant role in the high survival rates observed in this study [56].

To evaluate the impact of treatment on oral health-related quality of life (OHRQoL), the Oral Health Impact Profile (OHIP) is a valuable tool. The shorter version, OHIP-14, has proven more practical for clinical applications and is recognized as a reliable instrument in both clinical and epidemiological settings [13]. While the original OHIP survey provided a comprehensive assessment, which was deemed too time-consuming for routine clinical use, this led to the adoption of a more concise questionnaire. This version (OHIP-14) effectively evaluated multiple dimensions of patients’ lives before and after treatment [13,57,58]. 

The OHIP-14 has shown good internal consistency and construct validity, correlating well with other measures, such as the Visual Analog Scale (VAS) for pain and xerostomia [59,60]. Compared to other instruments like the Geriatric Oral Health Assessment Index (GOHAI), the OHIP-14 appears to be more sensitive to changes in psychological and behavioral outcomes, making it more effective in evaluating treatment responses [61]. These findings support the use of OHIP-14 as a preferred measure over VAS in assessing oral health-related quality of life.

According to the literature, PMMA interim prostheses have shown significant improvements in oral health-related quality of life (OHRQoL), particularly in areas such as functional limitations, psychological discomfort, and social disability [13]. PMMA’s favorable properties—such as its lightweight nature, esthetic appeal, and sufficient mechanical strength for interim use—contribute to patient satisfaction during the healing phase [1]. 

Compared to protocols using metal–acrylic or composite resin materials, milled PMMA prostheses demonstrate superior esthetic outcomes due to their translucency and ease of customization [37]. Additionally, PMMA’s cost-effectiveness and rapid fabrication via CAD/CAM workflows make it more accessible, reducing patient stress associated with prolonged treatment times [12]. Although rigid materials like monolithic zirconia provide greater long-term durability, their higher weight and cost can negatively affect patient perception in interim stages [19]. 

A notable strength of this study is the consistency maintained in both the treatment protocol and patient demographics. These results are especially encouraging for high-risk patients, such as those with a history of periodontal disease. Additionally, the use of PMMA as the material for interim prostheses reinforces its suitability for immediate-loading protocols [11,37]. 

PMMA’s favorable mechanical properties, such as high flexural strength and fracture resistance, combined with its cost-effectiveness and ease of fabrication, make it an optimal choice for immediate restoration. These attributes not only ensure stability and functionality during the healing phase but also contribute to improved patient satisfaction and predictable outcomes in high-risk clinical scenarios.

## 5. Conclusions

This study suggests that treating edentulous patients with immediate functional loading complete-arch implant-supported PMMA interim prostheses using a fully digital workflow is a successful treatment option. However, further research with longer-term follow-up is necessary to fully validate these findings and ensure their long-term efficacy.

## Figures and Tables

**Table 1:** Clinical variables.

Variable	Level	Number	Percentage
Sex	Male	36	50
	Female	36	50
Range of age	<45 years old	54	12.5
	Between 45 and 60	168	38.88
	>60 years old	210	48.61
Smoking habit	Smokers	24	33.3
	Non-smokers	48	66.7
Location	Maxilla	37	51.4
	Mandible	35	48.6
Implant diameter *	3.75	39	9.02
	4.3	275	63.65
	5	118	27.31
Abutment *	1.5	29	6.71
	2.5	286	66.2
	3.5	104	24.07
	Angulated	13	3

* Implant diameter and abutment height are in millimeters (mm).

**Table 2:** Mean marginal bone loss (MBL) related to variables.

Variable	Level	Mean MBL *
Sex	Male	−0.432 ± 0.036
	Female	−0.272 ± 0.038
Range of age	<45 years old	−0.257 ± 0.074
	Between 45 and 60	−0.363 ± 0.038
	>60 years old	−0.377 ± 0.043
Smoking habit	Smokers	−0.392 ± 0.045
	Non-smokers	−0.335 ± 0.033
	Resin	−0.798 ± 0.099
Opposing	Teeth	−0.366 ± 0.050
dentition	Ceramic	−0.335 ± 0.099
	Zirconia	−0.300 ± 0.033

* Mean MBL was measured from radiographs made post-surgery (T0) and at the 3-month follow-up examination (T1).

## Data Availability

The data that support the findings of this study are available on reasonable request from the corresponding author. The data are not publicly available due to privacy or ethical restrictions.
